# Multi-Blockchain-Based IoT Data Processing Techniques to Ensure the Integrity of IoT Data in AIoT Edge Computing Environments

**DOI:** 10.3390/s21103515

**Published:** 2021-05-18

**Authors:** Sung-Ho Sim, Yoon-Su Jeong

**Affiliations:** 1 College of General Education, Semyung University, Jechon-si 27136, Korea; shshim@semyung.ac.kr; 2 Department of Information Communication Engineering, Mokwon University, Daejeon-si 35349, Korea

**Keywords:** AIoT edge computing, multiple blockchain, integrity of IoT data, synchronization using location information, cross-distributed and blockchain linkage processing

## Abstract

As the development of IoT technologies has progressed rapidly recently, most IoT data are focused on monitoring and control to process IoT data, but the cost of collecting and linking various IoT data increases, requiring the ability to proactively integrate and analyze collected IoT data so that cloud servers (data centers) can process smartly. In this paper, we propose a blockchain-based IoT big data integrity verification technique to ensure the safety of the Third Party Auditor (TPA), which has a role in auditing the integrity of AIoT data. The proposed technique aims to minimize IoT information loss by multiple blockchain groupings of information and signature keys from IoT devices. The proposed technique allows IoT information to be effectively guaranteed the integrity of AIoT data by linking hash values designated as arbitrary, constant-size blocks with previous blocks in hierarchical chains. The proposed technique performs synchronization using location information between the central server and IoT devices to manage the cost of the integrity of IoT information at low cost. In order to easily control a large number of locations of IoT devices, we perform cross-distributed and blockchain linkage processing under constant rules to improve the load and throughput generated by IoT devices.

## 1. Introduction

Recently, the growing interest in the Fourth Industry Revolution and AI (Artificial Intelligence) has led to the need for Artificial Intelligence of Things (AIoT) in a variety of smart environments such as commercial surveillance, autonomous driving, and robots [1]. However, the computing resources of AIoT devices do not meet the requirements for model prediction accuracy and real-time responses. Existing cloud computing uses offloading of some computationally intensive tasks to remote cloud servers to improve the prediction accuracy of information transmitted and received from AIoT applications [2].

AIoT edge computing refers to a computing environment that combines IoT and artificial intelligence systems [3]. Instead of processing data in the cloud of a powerful central computer server, some data crunching and algorithms currently run on the edges of several devices, such as video cameras with improved processor performance. Powerful devices analyze a small number of data, but a small number of data can usually be analyzed on many computers. Some of the benefits of this network architecture can be significantly reduced cloud service costs due to increased latency, improved data privacy, and reduced cloud resources used.

AIoT edge computing environments should be able to guarantee large-scale AIoT network delays and data protection, but AIoT computing environments currently operating require new solutions that reflect AIoT characteristics as information is being processed around the cloud [4]. In particular, information collected from IoT devices can be given different access policies between AIoT networks through prior analysis and prediction in AIoT to minimize latency by pretreatment of information control and accessibility in AIoT computing environments. However, applications related to IoT devices are exposed to serious security risks due to the lack of clear security measures in place compared to the rate at which IoT services evolve. This shows that methods of protecting shared data between IoT devices are recognized as very important, as most IoT data are computationally exchanged between proposed devices in untrusted wireless environments.

To efficiently manage heterogeneous IoT information in AIoT edge computing, IoT information must be continuously generated and transmitted over wired and wireless environments [5]. At this time, to ensure the integrity of IoT information, security levels must be guaranteed at no additional computational cost so that IoT information can be compromised multiple times.

Recent work has focused on recognizing nodes that make up a cluster or generating group keys based on group members that serve as servers for IoT information security. In particular, in the field of IoT information protection, PKI techniques using password-based recognizers [6] to ensure safe data processing and archiving between heterogeneous IoT devices in AIoT edge computing environments, applying group signatures to prevent data from maliciously manufactured IoT devices [7] and techniques [8].

In AIoT edge computing environments, blockchain has great potential to solve various problems related to IoT [9]. Users who use blockchain, which is considered one of the disruptive technologies in various fields (economy, society, etc.), including the IT field, can trade with each other without a central server and maintain a consistent ledger, so all it needs to do is to solve the problem of distributed transmission, calculation, and storage.

In this paper, we propose a blockchain-based IoT big data integrity verification technique optimized for AIoT edge computing environments. The proposed technique minimizes IoT information loss by linking information and signature keys of heterogeneous IoT devices distributed in AIoT edge computing environments with blockchain-based multiple hashchains. Furthermore, each block of IoT information can be effectively applied in various AIoT edge computing environments by storing hash values of previous blocks and linking chains to previous blocks. The proposed technique is made reliable by verifying the received blocks and determining whether to add distributed information linked to the blockchain, thereby verifying the hash values stored in the blockchain.

Proposed techniques have the following objectives to manage the information integrity of IoT devices at a low cost. First, in line with changes in AIoT edge computing environment, we maintain geographic information-based IoT location information to maintain trust and consensus between central servers and IoT devices to sustain synchronization. Second, it dynamically provides security according to the situation information of IoT devices operating in AIoT edge computing environments. Third, to easily control numerous IoT devices of heterogeneous species, access to IoT devices is improved through a multi-step approach of hierarchical structure. Fourth, the proposed technique is distributed so that the IoT information can be linked to a blockchain to improve the load and processing rate that can occur between IoT devices. Fifth, the proposed technique minimizes the processing time of IoT devices requesting AIoT edge computing services because the layer-distributed IoT devices are cross-distributed and processed according to certain rules.

The remainder of this paper is organized as follows. Section 2 explores the blockchain and existing research. Section 3 proposes IoT big data integrity verification techniques based on blockchain optimized for AIoT edge computing environments, and Section 4 evaluates the performance of proposed and existing technologies. Finally, we conclude in Section 5. 

## 2. Preliminaries

### 2.1. AIoT Edge Computing

Edge computing performs computing at or near the physical location of a user or data source. Processing computing services in a location close to the device they use allows users to receive faster and more reliable services and allows companies to benefit from flexible hybrid cloud computing. Edge computing handles use cases (virtual/augmented reality, big data analytics, and collection on-site, recoverability, and cost savings) that cannot be adequately addressed by a centralized approach to cloud computing, mainly due to networking requirements or other constraints. Edge computing can not only reduce data load and reduce processing time, but also respond much faster than cloud computing environments. In other words, cloud computing is suitable in environments where the amount of data generated by the terminal is small and the transfer delay is not fatal, but edge computing is essential in environments where real-time processing is of high importance.

IoT Edge is software that extends data processing and machine learning capabilities in the cloud to end devices such as gateways and cameras, which can make Internet of Things applications smarter, safer, and more reliable. IoT Edge operates on Android Things or Linux-based devices, with the following main components. First, runtime for gateway-class devices can have at least one CPU store, translate, process, and intelligence-inducing data obtained at the edge and interact with the rest of the cloud IoT platform. Second, edge IoT cores connect edge devices to the cloud more securely, and manage software or firmware updates and data exchanges with cloud IoT cores. Third, tensorflowlight-based edge ML runtime performs local machine learning inference using pre-trained models, which reliably reduces delay and enhances the flexibility of edge devices.

AIoT Edge Computing has been developed to handle the proliferation of Internet of Things devices, resulting in a proliferation of data volumes [3]. Cloud computing has been spotlighted by many companies, providing computing services such as servers, repositories, software, and analytics over the Internet. Although the number of users using cloud services has increased exponentially in recent years, there is no problem with the throughput of cloud servers and data centers because the cloud can provision more resources. Due to these environmental changes, the requirements for solving security problems in the communication process as well as the data delay that occurs in the process of analyzing and transmitting collected data are increasing. AIoT edge computing has seen a surge in the number of data as IoT devices have become popular in earnest, which has pushed cloud computing to its limits. To compensate for this, edge computing technologies have been developed, and instead of sending all data to the cloud for analysis, critical data are processed in real time. Edge computing can guarantee three things: data load reduction, security, and fault response [2]. Looking at the three guaranteed features of AIoT edge computing in detail, first, data load reduction can minimize the load because it only handles the data generated by the corresponding IoT device. Second, security is relatively good compared to cloud computing because security handles data collection and processing on its own. Third, failure response is catastrophic when servers are paralyzed when using cloud computing, but edge computing performs its own computing, which can effectively respond to failures.

AIoT is a technology that combines Internet of Things technology with artificial intelligence technology, which means convergence technology that develops, mounts, and utilizes artificial intelligence according to the environment and characteristics in which IoT is used [4]. In particular, AIoT is a key convergence technology (DNA, Data, Network, AI) that expands connectivity to virtual space and implicitly expands intelligence from form to super-convergence and revolutionizes the industry.

AIoT’s implementation method is largely divided into using cloud intelligence or mounting intelligence on objects. Table 1 shows examples that can be implemented by dividing AIoT implementation method into cloud intelligence utilization and object intelligence.

AIoT requires analytical capability (intelligent filtering) to evaluate data as quickly as data occurs and convert them into useful information that can be transferred into action. Because AIoT can derive new added value from the level of “intelligence” rather than “connection”, AIoT has the ability to exponentially increase business performance and revolutionize the industry when many data meet AIoT.

### 2.2. Blockchain

In recent years, 3D printers, big data, robotics, and artificial intelligence have been mentioned under the name of the Fourth Industrial Revolution. IT experts expect the blockchain to create synergy by weaving new technologies together. The blockchain technology that appeared with Bitcoin was very interesting, but it is not easy to understand [10].

Blockchain is a kind of uncentralized database that prevents forgery and tampering by verifying the data integrity of all transactions by all users of the network without a central administrator [11]. Distributed directors used in the blockchain are divided into licensed and open directors depending on whether or not data integrity is restricted from participating.

Open directors are characterized by the fact that certain individuals or organizations do not have distributed ledgers, unlike licensed ones. Therefore, any open director can add data to the ledger, and all participants with access to the ledger can have a copy of the ledger. This advantage prevents an open ledger from adding fraudulent or unauthenticated data because those with access must demonstrate validity when making changes.

Recently, several experts have examined the possibility of a blockchain, and the area that is making the most rapid change is Customer Relationship Management (CRM) using a closed-blockchain method [12,13]. Although interest in the blockchain has fallen slightly since regulations on Bitcoin investment were announced at home and abroad, new attempts based on the blockchain have been made steadily throughout the industry.

### 2.3. Related Works

In AIoT edge computing environments, we strive to maintain data integrity and security at all times. However, users of AIoT edge computing services want to identify problems with data corruption and deletion and take corresponding actions immediately. Blockchain technology is being offered as a new way to maintain a data integrity system.

K. N. Khaqqi et al., proposed solutions that could facilitate the adoption of long-term reduction technologies while reducing emissions production for Industry 4.0 integration [14]. This method combines blockchain technology with the Emissions Trading Scheme (ETS) to solve fraud problems and improve the effectiveness of ETS. Furthermore, this paper uses multiple criteria analysis to reduce emissions of existing ETS models by showing examples of the internal operation of a reputation-based trading system.

Z. Yang et al., proposed a blockchain-based reputation system to ensure the reliability of the data generated by the vehicle system [15]. After reaching an agreement on the vehicle rating, the system uses a message hash without an agreement to compare and verify the rating by the vehicle. However, the disadvantage of this system is that additional costs have not been assessed in terms of delay time to reach consensus.

A. Schaub et al., proposed a resource-based reputation system that preserves the benefits of anonymous ratings on assets by preserving consumers’ privacy [16]. However, the system makes it difficult to assess the overall capacity of token generation to separate the ratings of consumer transactions, and some grades have the disadvantages of being penalized because there is no direct connection between transactions and assaults.

A. Moinet et al., proposed a model to maintain a minimum level of confidence to reduce the damage to the wireless sensor network [17]. The downside is that the proposed model is to be written only at the network node level and lacks detailed reliability because services are not available on more than one node. The message digest used in the proposed model is used as the only criterion for authenticating the message.

In addition to the above studies, research related to IoT data security focuses on the clustering method of nodes responsible for a key generation between nodes in a cluster group [18,19,20,21,22,23,24,25,26].

A. Karati et al., proposed a PKI system cryptography for clusters to secure storage in a secure industrial IoT environment based on node ID or group membership [18]. This technique used signature cryptography to create a safe recognizer.

C. Esposito et al., proposed a group signing technique to prevent third parties from maliciously manipulating IoT data [19]. However, this technique reduces the inter-node communication overhead, but as the number of nodes increases, the communication overhead increases inefficiently [20].

Q. Bouachir et al., describe the knowledge and emerging issues of cyber-physical systems that fuse blockchain and fog computing to expand low-power, multi-functional sensing platforms that can monitor and communicate information in various fields, such as transportation, healthcare, and industry [21]. Additionally, this approach improves the quality of service (QoS) and experience (QoE) by performing data storage and processing tasks that are physically close to data sources in a distributed infrastructure.

G. Dong et al., proposes a framework to safely audit IoT data integrity based on the Hyperledger Fabric [22]. This technique uses the anonymity of the fabric itself while utilizing the characteristics of open and non-sensory information in the blockchain to ensure traceability. In particular, this technique complements the security problem of centralized TPA (third-party auditors) based on a consortium blockchain.

P. Urien proposes an algorithm to ensure the firmware integrity of IoT devices using bijective MAC timestamps [23]. The algorithm calculates the memory thumbprint of the hash function according to the pseudo-random order fixed by the permutation P.

Chen et al., proposes a blockchain-based data inspection method to protect IoT integrity [24]. This method ensures the data integrity of IoT networks through distributed data authentication of blockchain. However, IoT, with limited computing and network resources has problems that traditional blockchain systems cannot be directly applied.

Cherupally et al., proposed a lightweight and scalable Directed Acrylic Graph (DAG)-based distributed ledger for Lightweight and Scalable DAG-based distributed ledgers (LSDI) that can work with IoT GW(GateWay) to provide IoT data integrity verification quickly [25]. This technique guarantees high transaction throughput and scalability by eliminating older transactions, reducing the storage overhead, and computing overhead of IoT GW.

Kuo et al., proposed blockchain index storage to provide IoT supply chain tracking [26]. This method supported adaptive transaction arrival rates to control the latency and cost of demand, and the supply chain data was stored directly in the blockchain to prevent malicious data modulation. However, this method has the problem of significantly increasing latency and cost because it generates huge transactions and data for large-scale IoT devices.

## 3. IoT Data Integrity-Verification Techniques Optimized for Distributed Cloud Environments

With the recent advent of the Fourth Industrial Revolution, IoT technology has developed faster than other technologies, and many changes have been made to the cloud environment. In particular, cloud environments are often used to prevent loss and deletion for various reasons (such as natural disasters, network attacks, disk risks, administrator error handling, and errors in cloud storage services) stored on portable devices (smartphones, tablets, etc.).

This section proposes an optimized blockchain-based IoT data integrity verification method to maintain the integrity of the data to prevent problems that damage or delete the user’s data in a distributed cloud environment. The proposed technique ensures the integrity of IoT information by connecting IoT information and signature keys hierarchically with multi-hash chains to group IoT information into multiple groups. Furthermore, IoT information distributed in heterogeneous AIoT edge computing environments was verified with hash values stored in the blockchain to ensure the reliability of IoT information.

### 3.1. Overview

As IoT devices are used in various fields in AIoT edge computing environments, interest in the safety of information sent and received from IoT devices is increasing. Because information transmitted and received by IoT devices is transmitted in a wireless environment, safety problems are more serious than in a wired environment. Furthermore, in AIoT edge computing environments, cloud service support policies are supported differently depending on the rights of users accessing cloud services. To handle only certain kinds of information among different types of IoT information in AIoT edge computing environments, it is necessary to minimize information loss by managing additional information that can only recognize IoT information.

The proposed technique aims to support cloud policies on a blockchain basis so that users can receive cloud services smoothly by users’ cloud service requirements while ensuring the safety of information on IoT devices deployed in distributed cloud environments.

The proposed technique minimizes IoT information loss by tying each IoT information and the signature key placed in AIoT edge computing environment with multi-hashchains and managing them in block form. By doing so, the integrity of IoT information can be managed at low cost by storing the previous block of IoT information and the current block or the current block and the next block, respectively, as hash values. Furthermore, the proposed technique was designed to minimize the loss of information between IoT devices and maintain trust between IoT devices by maintaining long-term IoT synchronization so that IoT devices can be operated smoothly in AIoT edge computing environments. The proposed technique minimizes the processing time of the cloud service of IoT devices because they are cross-distributed by certain rules.

### 3.2. System Architecture

**Figure 1:**
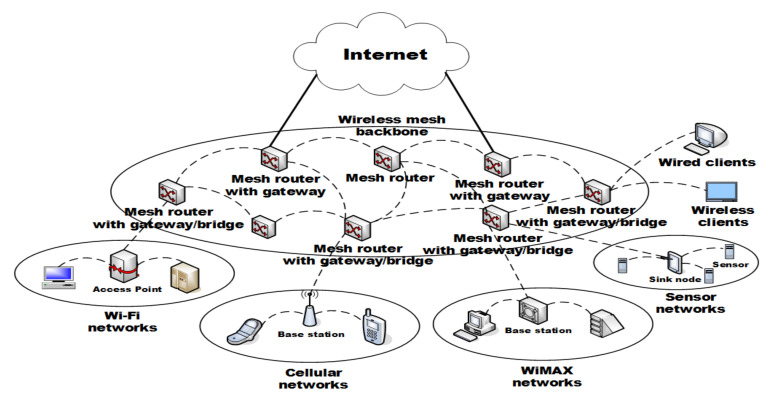
AIoT edge computing environments in proposed technique.

The proposed technique uses a network model consisting of servers, intermediate parties, and devices, as shown in Figure 1. Devices deployed in the network maintain their own position in the group while processing data. Devices consist of a cluster-based network, with an intermediate party and a device-to-device distance of 1 hop. Data collected from the device are collected by the intermediate party for primary collection, and the collected data are delivered directly to the server or to the adjacent intermediate party for delivery to the server. For intermediate parties away from the server, in the proposed technique, data collection within each group exploits a multipath hashchain. Credential values generated by multipath hashchains are sent to the server without being re-collected by other intermediate parties.

The proposed technique classifies IoT user attribute information hierarchically, which is probabilistically weighted according to the status information of IoT users. Weights allocated to IoT users are used to maintain synchronization between servers and IoT users at regular intervals, thus preventing leakage of privacy information by IoT users to third parties. In particular, the proposed technique validates synchronization with the server using stochastic weights of evaluation results by pairwise comparisons.

**Figure 2:**
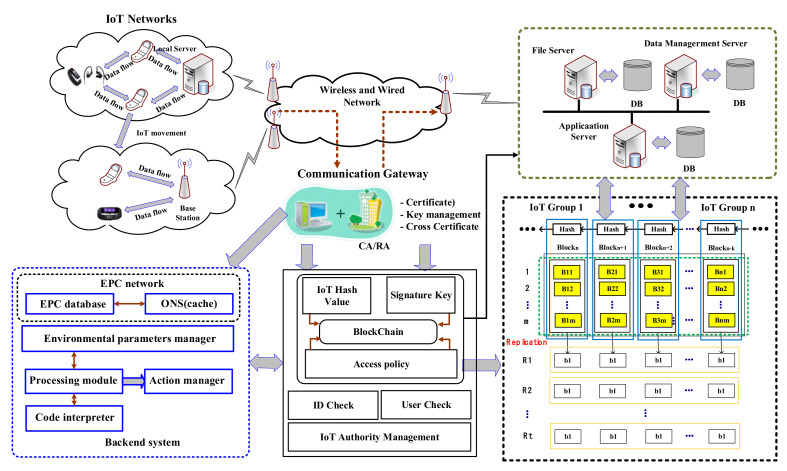
IoT data operation process of proposed technique.

Figure 2 shows a system model that can safely receive cloud services by connecting hash information (IoT information and signature keys) of IoT devices with multi-hash chains without any special policy support according to the access authority of IoT devices in distributed cloud environments. The proposed technique in Figure 2 treats IoT hash values and signature keys as blockchains among information on IoT devices, and IoT authority management and cryptographic policies are classified as non-blockchains.

The reason for this is that the proposed technique allows multiple distributed cloud networks to connect to a single core network flexibly through a point of access (AP). Assuming that all IoT devices distributed in a distributed cloud network have access to the AP located closest to the IoT device, the network, including the AP, has N IoT devices, and all APs can connect to the cloud as well as server administrators over the core network.

In Figure 2, the information of all IoT devices can be negotiated with the multi-hash function h: ${\left\{0,1\right\}}^{*}$ → ${\left\{0,1\right\}}^{k}$ so that it can be transmitted and received in a distributed cloud environment. The negotiated blockchain of IoT information is reproduced in any block $b_{i}$(= $h^{i}\left(s\right)$) (*i* ∈ Integer, $s\in_{R}{\left\{0,1\right\}}^{i}$) size of k-bit, where *i* stands for security parameters and s stands for seed.

The reason why any block $b_{i}$ is created with k bits in the proposed technique is to simply classify and manage complex information sent and received from IoT devices. This is why the proposed technique assumes that cloud services are possible through a blockchain for data generation and management. Furthermore, the proposed technique can present the association and similarity of IoT information as each block category of IoT information through the distribution of Bernoulli by binding any block $b_{i}$ of k bits into a blockchain.

### 3.3. Measuring IoT Data Synchronization Using IoT Location Information

**Figure 3:**
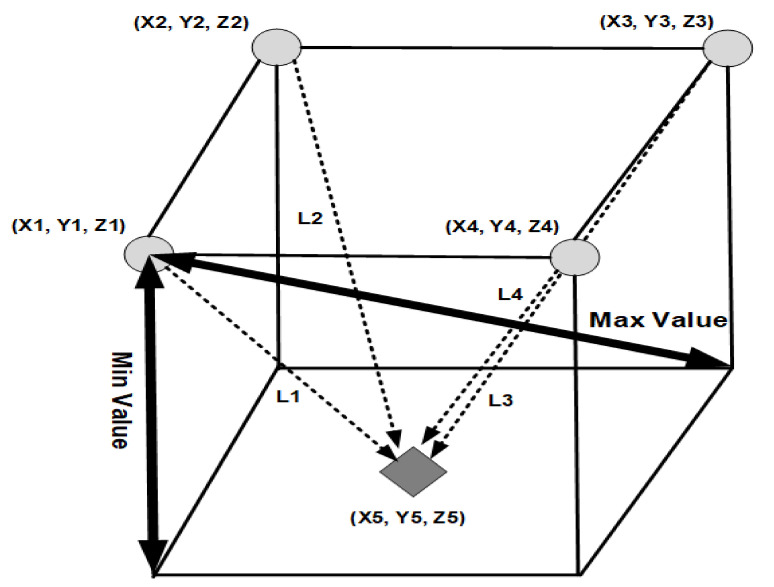
Measure integrity synchronization of IoT data using AIoT location information.

In the proposed technique, we use the Thomas algorithm’s multivariate method as shown in Figure 3 to describe the minimum and maximum values of distances that can occur within the space for measuring AIoT position information, while simultaneously correcting the accuracy.

In the proposed technique, the location of IoT devices is calculated using Equation (1) to keep IoT localization-based data keys efficient while minimizing errors in IoT information located in AIoT edge computing environments.

(1) $$L_{1}^{2}={\left(X5-X1\right)}^{2}+{\left(Y5-Y1\right)}^{2}+{\left(Z5-Z1\right)}^{2}\;\;L_{2}^{2}={\left(X5-X2\right)}^{2}+{\left(Y5-Y2\right)}^{2}+{\left(Z5-Z2\right)}^{2}\;\;L_{3}^{2}={\left(X5-X3\right)}^{2}+{\left(Y5-Y3\right)}^{2}+{\left(Z5-Z3\right)}^{2}\;\;L_{4}^{2}={\left(X5-X4\right)}^{2}+{\left(Y5-Y4\right)}^{2}+{\left(Z5-Z4\right)}^{2}\;\;$$

As shown in Figure 3, IoT devices deployed in AIoT edge computing environments establish full pairwise keys (FPs) to each other using the location information in expression (1), and random pairwise keys based on predefined probabilities with adjacent IoT devices and communication ranges of randomly selected IoT devices. The reason for this setting is that it is highly resistant to deployment errors for IoT devices and can achieve high key connectivity.

In the proposed technique, the location information $\mathrm{LI}\left(x\right)$ of IoT devices deployed in AIoT edge computing environments can verify the integrity of the location information of IoT devices by sampling and distributing topographical information of IoT devices using N*-1th* polynomials, such as expression (2).

(2) $$\mathrm{LI}\left(x\right)=\left\{\begin{array}{cc} a_{1}+a_{2}x^{1}+\cdots +a_{n}x^{n-1}\;mod\;n & ,\;if\;a,\;n>1\\ 1 & ,\;otherwise \end{array}\right.$$

where n means the number of devices in IoT devices, and a means the magnitude of the location information in IoT devices.

In Equation (2), $x_{1}$, $x_{2}$,⋯, and $x_{n}$ are made of n different datasets, such as Equation (3).

(3) $$\left|\begin{array}{cc} \begin{array}{cc} \begin{array}{cc} 1 & x_{1} \end{array} & x_{1}^{2}\\ \begin{array}{cc} 1 & x_{2} \end{array} & x_{2}^{2} \end{array} & \begin{array}{cc} \cdots & x_{1}^{n-1}\\ \cdots & x_{2}^{n-1} \end{array}\\ \begin{array}{cc} \begin{array}{cc} \vdots & \vdots \end{array} & \vdots \\ \begin{array}{cc} 1 & x_{n} \end{array} & x_{n}^{2} \end{array} & \begin{array}{cc} \ddots & \vdots \\ \cdots & x_{n}^{n-1} \end{array} \end{array}\right|$$

Based on the above, the proposed technique is represented by Equation (3) in the form of a product of ($x_{j}-x_{i}$) to generate keys in the subnet to protect IoT information in AIoT edge computing environments.

### 3.4. Generating Multiple Blockchains Based IoT Information

All IoT devices distributed in AIoT edge computing environments generate and send IoT information based on blockchain to IoT devices other than final destination devices. At this time, the proposed technique assumes that the IoT information is bigger than the number of IoT devices. All IoT devices distributed in AIoT edge computing environments use ${}_{R}{\left\{0,1\right\}}^{n}$ to create an arbitrary block in the form of n bits as shown in Equation (4) and then perform Equation (5) to replicate each block created.

(4) $$B_{\left(n,b\right)}=\{\left(n,b\right)|b=1,2,3,\ldots ,m\}$$

(5) $$H_{x}\left(ID_{k}\right)=RB_{\left(x,b\right)}=\{\left(x,b\right)|x=\mathrm{Replication}\;number;b=Block\;number\}$$

Equation (5) enables continuous hash chain processing without loss of information on adjacent IoT devices when two hash values are configured according to sequence number i and number of applications of odd/even IoT information.

The proposed technique will verify the integrity of the IoT information by adding the two hash values generated by the number of applications of odd/even to the first and last of the multiple hash chain. Such a method can lower overhead and then verify the integrity of existing IoT information through signature, so it can be optimized for an AIoT edge computing environment in IoT information generation. Algorithm 1 shows an algorithm for generating IoT information sent and received from IoT devices distributed in AIoT edge computing in a block form by combining them with multiple hash chains.

The IoT information generated by Algorithm 1 uses polynomial properties such as $\left(\begin{array}{c} n\\ k \end{array}\right)$=$\left(\begin{array}{c} n-1\\ k-1 \end{array}\right)$ + $\left(\begin{array}{c} n-1\\ k \end{array}\right)$ to compare the important information of IoT information $a_{nk}$ = $\left(\begin{array}{c} n-1\\ k-1 \end{array}\right)$ with the relation components of $a_{ij}$ defined as $a_{j}$ relatively more important than $a_{i}$. A zero correlation information between $a_{xy}$ and $a_{yx}$ implies no importance between pairwise comparisons matrices.

**Algorithm 1.** IoT information generation algorithm based on blockchain.1: Initialize information on IoT distributed across AIoT edge computing.2: **for all** IoT information do3:    **if**  Receive IoT Information from IoT Devices then4:        Compare IoT Information received from other IoT Devices5:        Store the IoT Information block6:        **if** the IoT Information block can be identified then7:          Generate random blocks in n-bit form using
${}_{R}{\left\{0,1\right\}}^{n}$ from all IoT Information.8:          Convert each block to replication9:          **if** Generate hash values for odd/even10:             Add to first and last of multiple hash chains11:             Verification of the integrity of IoT Information12:         **else**13:             Regenerate hash values for odd/even14:         **end if**15:       **else**16:         Reconfirm the IoT Information17:       **end if**18:    **else**19:       Request IoT Information received from other IoT Devices20:    **end if**21: **end for**22: **return** creating replication information for Add/Even

By Algorithm 1, the proposed technique groups IoT information to arbitrary sizes along with weights, and then stores IoT information and signature keys in a blockchain with access control policies. However, IoT information and computational-intensive information other than signature keys are treated with non-blockchain.

**Figure 4:**
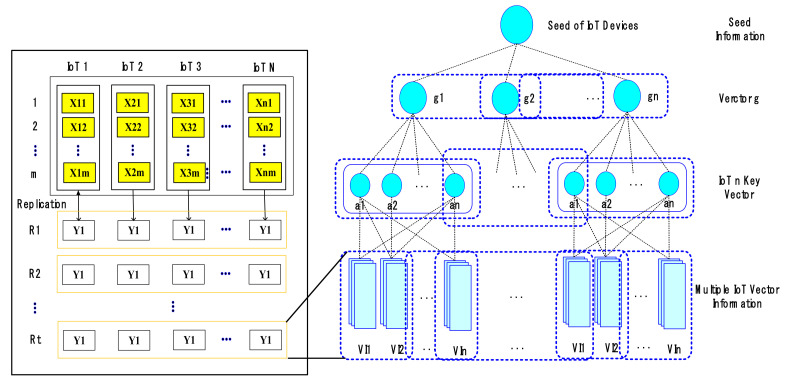
IoT block information grouped with multiple hash chains.

The proposed technique uses IoT block information grouped with multiple hash chains, such as Figure 4, to minimize interference between IoT information in the process of processing large amounts of information.

To accurately handle large-scale IoT block information by linking IoT block information to each other, such as Figure 4, we allow IoT block information $b_{t}$ to select a signature key seed $\hat{y_{t}}$ according to similar information $h_{t}$ of IoT block information. At this time, IoT block information is grouped by ranking with high relevance of IoT block information in the process of IoT block information path and core extraction. With the proposed technique, we examine the similarity of IoT block information to the connected group and apply polynomial secret keys of *N* − 1 order when grouping the possible whole paths into one group. This is because it is possible to predict and analyze patterns in IoT block information by finding additional paths through highly relevant pattern analysis.

### 3.5. Blockchain-Based IoT Hash Information Connection

**Figure 5:**
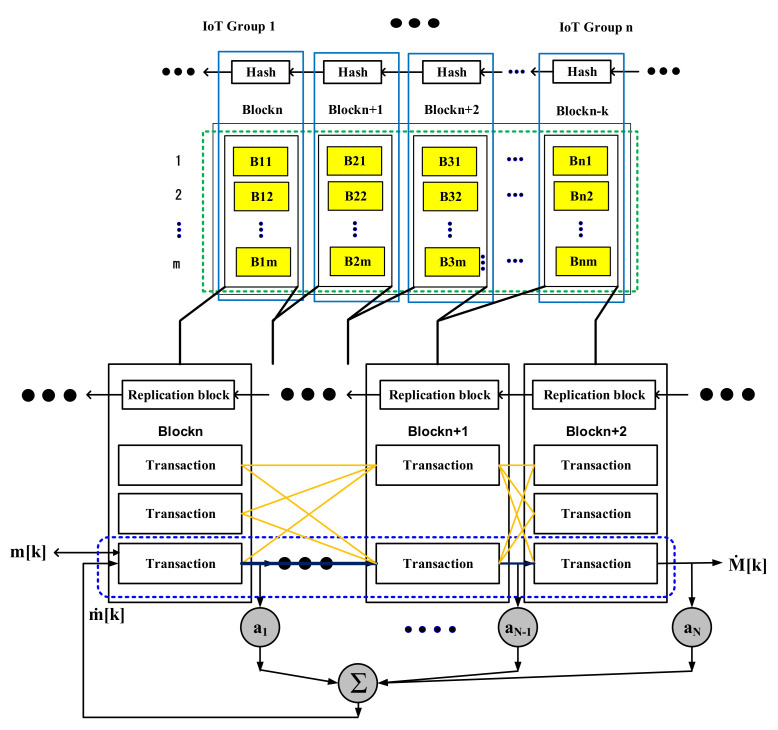
Blockchain-based multi-data connection structure.

Blocks of IoT information generated by replication give weight to each hash chain with a certain probability of multiple tie-ups hierarchically. At this time, IoT information included in lower-level groups should be reconstructed periodically according to the nature of IoT information, such as Figure 5, to be organized into hierarchical multi-level groups.

The proposed technique can minimize delays in processing IoT information processed in real-time by adding two hash values to the first and last of multiple hash chains among information sent and received from IoT devices.

When the k-th IoT information block is $\dot{m}\left[k\right]$ like Figure 5, the error of the IoT information block is as predictable as $d\left[k\right]$(= $m\left[k\right]$ − $\dot{m}\left[k\right]$. However, the proposed technique will generate IoT information block $m\left[k\right]$ by adding error block $d\left[k\right]$ by estimating any block $\dot{m}\left[k\right]$ among IoT information blocks through the same process as Figure 4. Unlike conventional techniques, the proposed technique configures the number of comparisons as many times as n(*n* − 1)/2 to minimize connection errors in IoT information blocks because large amounts of information can be generated by servers for any t-hour in AIoT edge computing environment. Algorithm 2 shows algorithms for connecting IoT information blocks with multiple merkle trees.

**Algorithm 2.** IoT Information Block Hash Connection.1: initialize IoT information block 2: **while** IoT information block > 0 **do**
3:    Generate random blocks in n-bit form using ${}_{R}{\left\{0,1\right\}}^{n}$4:    Server checks its blocks 5:    **if** Replication block data present in blocks  then6       Makes merkle tree in republication block7:       Calculates block hash from merkle tree8:       Inform other IoT devices9:    **else**10:      Request replication block data11:    **end if**12: **end while**
13: **return** IoT devices generates the new block 

In Algorithm 2, the proposed technique can carry out error judgment on large-capacity IoT information blocks through connection error of IoT block information, thus improving the connection accuracy of IoT block information.

### 3.6. Connection Renewal of IoT Information

To update the connection of IoT information distributed in AIoT edge computing environments, the probability of connection of IoT information will be updated periodically through probability values such as Equation (6) for hash-chained information according to the type, function, and characteristics of IoT information.

(6) $$\mathrm{Prob}=\left\{\begin{array}{cc} \frac{n}{L}-\frac{n{\left(\left(L-1\right)!\right)}^{2}}{L^{2}\left(L-21\right)!\left(L-1\right)!} & \left(L\;\geq K\right)\\ \frac{n}{L} & \left(1\leq L\leq K\right) \end{array}\right.$$

where L means the length of the hash chain and n means the number of IoT information.

The probability value of IoT connection, such as Equation (6), will be randomly grouped or subnets created to suit the IoT characteristics when extracting and detecting a large amount of IoT information sent and received in the AIoT edge computing environment. Proposed techniques update n blocks of high similarity to block probability values, such as wth Equation (7), through connection renewal of IoT information distributed in AIoT edge computing environments.

(7) $$P\left(B’\right)=\sum_{i=1}^{n}P\left(B’|B_{i}\right)P\left(B_{i}\right)$$

In Equation (7) the probability value $P\left(B_{i}\right)$ that IoT information block $B^{\prime }$ will occur among the total IoT information blocks is obtained by multiplying $P(B^{\prime }|B_{i})$ by the probability value $P\left(B_{i}\right)$ when a specific IoT information block $B^{\prime }$ is extracted. As a result, the conditional probability of $P(B_{i}|B^{\prime })$ will be included in the update of the similarity of IoT information. The proposed technique is calculated with Equation (8) for the approximate value T to become an equal equation, no longer an approximation, when n multi-dimensional block information $b_{1}$, $b_{2}$,⋯, and $b_{n}$ is used to update the similarity.

(8) $$T_{i}=\frac{<T,\;b_{i}>}{<b_{i},\;b_{i}>}=\frac{1}{{\left|\left|b_{i}\right|\right|}^{2}}<T,\;b_{i}>\;\;\;I\;=\;1\;,\;2,\ldots ,\;n$$

where ${\left|\left|b_{i}\right|\right|}^{2}$ must have the same conditions as in Equation (9) to obtain $T_{i}$, which is orthogonal to each other.

(9) $$<b_{m},\;b_{n}\;>=\left\{\begin{array}{ccc} 0 & & i\neq j\\ {\left|b_{m}\right|}^{2} & & i=j \end{array}\right.$$

## 4. Evaluation

This section compares and evaluates the integrity of the blockchain-based IoT information in AIoT edge computing environment.

### 4.1. Environment Setting

**Figure 6:**
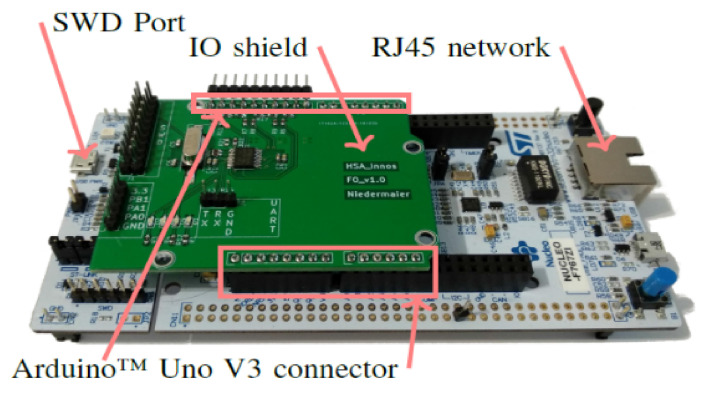
IoT device used in performance evaluation.

The configuration for evaluating the performance of the proposed technique is as shown in Table 2. It is assumed that IoT devices used in the proposed technique have an environment to transmit and receive IoT information using IoT Arduino equipment, such as in Figure 6, and that each AP’s base station consists of a multi-mesh network. Furthermore, each base station has servers collect and analyze the collected information in real time so that IoT information sent and received from antennas could be sent and received by equipment such as in Figure 6.

The experimental environment is constructed in a hierarchical structure using components of Table 2 (server, AIoT, IoT, etc.). The entire network is divided into 20 subnets, with servers (datacenter) at the top, so that each subnet is equipped with AIoT, which collects and processes data. Additionally, the number of IoT assigned to each subnet is 15.

The network scope of the performance evaluation is set at 500 m considering the range of IoT transmission and reception, and the bandwidth $\beta_{S}$/$\beta_{IoT}$ of the server and IoT is set at 10 MHz/5 MHz. Among the settings related to the blockchain, the limit for the maximum size of the blockchain is set at 0.5 to 2 Mbytes. The limit time δ required to reach the variable compensation coefficient is set to 0.01 s/KB. Other configuration settings are as shown in Table 2.

### 4.2. Performance Metrics

The performance metrics used in the evaluation of the proposed technique are evaluated by the integrity of IoT data and the latency handled by the AIoT and the server serving as gateways [27]. When IoT data is passed to AIoT, we obtain it like expression (10) using probability p according to whether or not the data to be processed in AIoT are stateless. Expression (10) can not only extend IoT data processed in AIoT to n-th hierarchies but also ensures reliability. Since Expression (10) gives probability information to IoT collection information processed in AIoT, similar information in IoT data can be selected (or bound) according to priority.

(10) $$X=\left\{\begin{array}{ccc} 1 & ,\;with\;probability\;p & \\ & & 0\leq p\leq 1\\ 0 & ,\;with\;probability\;1-p & \end{array}\right.$$

where probability p refers to the probability used in data processing in AIoT. The probability p updates the probability p by determining the state of the information passed by the IoT device.

Using expression (10), AIoT can define the processing probability of the data as expression (11).

(11) $$P(X=x|p)=p^{x}{\left(1-p\right)}^{1-x},\;\;x=0,1$$

The efficiency of IoT information processing in AIoT is obtained by expression (12),

(12) $$Efficiency_{AIoT}=\frac{\sum_{j=1}^{m}Processed\;Data\;of\;IoT_{j\;}}{\sum_{i=1}^{n}Total\;IoT\;Data\;number_{i}}\;\;=P\left(X=x|m,p\right)=\left(\begin{array}{c} m\\ y \end{array}\right)p^{y}{\left(1-p\right)}^{m-y},\;y=0,\;1,\;2,\ldots ,\;m$$

where m means the size of IoT data processed by AioT and p means the probability of data processed normally by AIoT.

In AIoT edge computing environments, latency can vary depending on the network environment. In the proposed technique, the latency of AIoT in the AIoT edge computing environment is $T_{AIoT}$, the latency of IoT devices comprising subnets is $T_{IoT}$, and the latency of AIoT edge computing network is $T_{Net}$, which is equivalent to Total Delay Time (TDT) silver expression (13).

(13) $$\mathrm{TDT}=T_{IoT}+T_{Net}+T_{AIoT}\times P+T_{AIoT}\times \left(1-P\right)$$

where P represents the probability of failure in AIoT acting as a subnet gateway server.

Assuming that each IoT data is transferred to AIoT without loss, the average processing latency (AD) of AIoT can be obtained, as shown in Equation (14).

(14) $$\mathrm{AD}=\frac{\left(IoT_{R_{1}}-IoT_{S_{1}}\right)+\left(IoT_{R_{2}}-IoT_{S_{2}}\right)+\cdots +\left(IoT_{R_{n}}-IoT_{S_{n}}\right)}{n}=\frac{1}{n}\left(\sum_{i=1}^{n}IoT_{R_{i}}-\sum_{i=1}^{n}IoT_{S_{i}}\right)$$

where n means the size of IoT data. $IoT_{S_{i}}$ represents the timestamp in which the *i*th data are transmitted, and $IoT_{R_{i}}$ represents the timestamp in which the *i*th data is received.

Equation (14) shows that after simply adding a timestamp of IoT data to AIoT, the timestamp of the data sent to AIoT is subtracted, and the timestamp of all IoT data is stored as a sum. However, in the proposed technique, the data loss probability P(= 0.05) is applied to expression (14) to obtain latency. The proposed technique maintains two bucket arrays to store timestamps for handling $IoT_{S_{i}}$ and $IoT_{R_{i}}$ separately and another two bucket arrays for maintaining $IoT_{S_{i}}$ and $IoT_{R_{i}}$, respectively.

### 4.3. Performance Analysis

#### 4.3.1. Evaluation of IoT Integrity Verification Time by Blockchain Generation Probability Value

**Figure 7:**
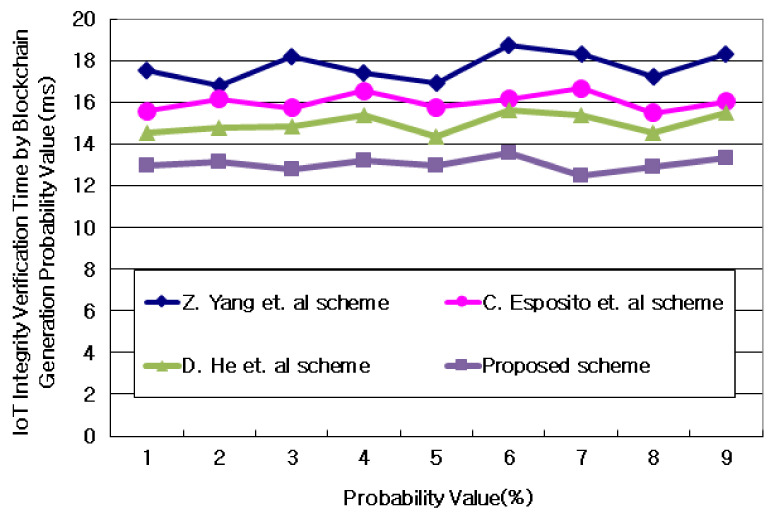
Time to verify IoT integrity based on the probability value of creating a blockchain.

Figure 7 and Table 3 show the results of comparing the verification time of the integrity of IoT information according to the probability value of creating a blockchain. According to Figure 7 and Table 3, the total IoT information according to the number of attribute information (date, time, size, purpose, etc.) of IoT information was grouped into n groups and the IoT information was linked to each other as probability values. Depending on the probability value of creating a blockchain, the verification time of IoT integrity according to the time to create an IoT blockchain improved by an average of 13.7% compared to the time when it did not. This result is because IoT information generated blocks of a certain size through sequential hierarchies and processed the linkage between IoT information. Furthermore, the proposed technique is because IoT information was accurately generated with a certain probability over time.

#### 4.3.2. Evaluating the Efficiency of IoT Information Processing in Subnet Gateway Servers

**Figure 8:**
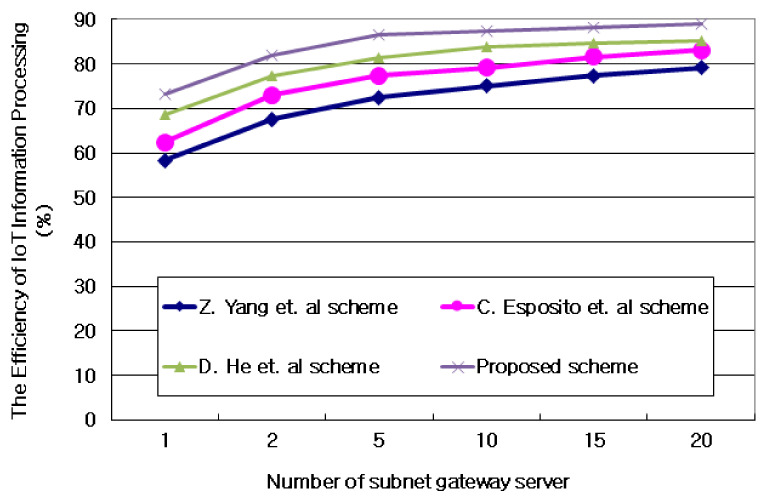
Evaluating the efficiency of IoT information processing in subnet gateway servers.

Figure 8 and Table 4 show an evaluation of the efficiency of information processing of IoT devices in the gateway server managing grouped subnets after grouping IoT devices distributed in AIoT edge computing environments into hierarchal structures. Information on IoT devices grouped into subnets is linked on a blockchain basis, so the performance of subnet gateway servers can vary depending on the density and scope of IoT information. As a result of the experiment shown in Figure 8 and Table 4, the proposed technique resulted in up to 15.1% improvement in the efficiency of the subnet gateway server compared to the case where IoT information is linked to a blockchain depending on the size of the subnet. These results are because the proposed technique was treated with a block hash chain to select a seed according to the probability value of the information linked to the IoT information contained in the subnet. These results are due to the reduced IoT information error rate between the *n* − 1 layer and the *n* + 1 layer by synchronizing IoT information using weights on probability information of IoT information.

#### 4.3.3. Delay Time for Verification of the Integrity of Blockchain Information

**Figure 9:**
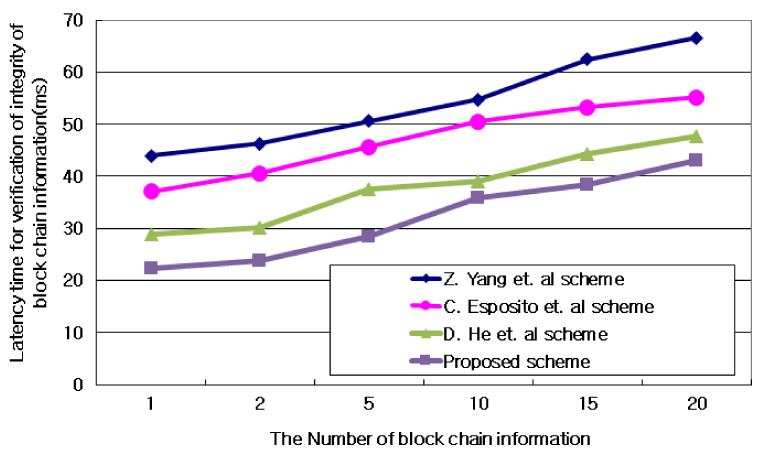
Delay time for verification of the integrity of blockchain information.

Figure 9 and Table 5 show a comparative evaluation of the delay that occurs when the subnet gateway server verifies the integrity of IoT blockchain information for IoT devices contained in the subnet after being grouped into n subnets in AIoT edge computing environment. Since IoT information linked to the blockchain is managed by grouping similar information hierarchically, the integrity of IoT information is verified according to the probability value of linking IoT information. At this time, as the block size of IoT information increased based on the blockchain, the proposed technique resulted in an average 7.9% lower integrity verification delay. These results are due to the creation of the IoT information into the blockchain so that the probability weights of the IoT blockchain are automatically linked to different probability factors. At this time, IoT information within groups shared by the blockchain was excluded from the creation of the blockchain.

#### 4.3.4. Overhead of IoT Integrity Validation by a Number of Subnet Gateway Servers

**Figure 10:**
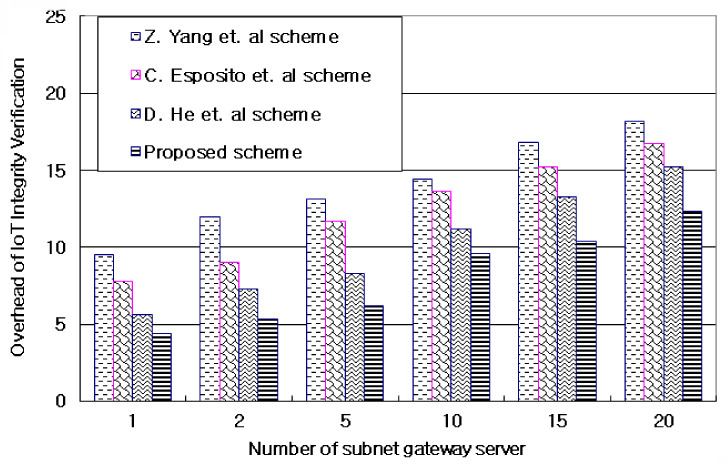
Overhead of IoT integrity validation by number of subnet gateway servers.

Figure 10 and Table 6 evaluates the overhead that occurs in verifying the integrity of grouped IoT information by the number of subnet gateway servers distributed in AIoT edge computing environments. The proposed technique can have different overheads depending on the amount of blockchain-based IoT information processing sent and received in a hierarchically grouped subnet depending on the connection level of IoT information. According to Figure 10 and Table 6, as the amount of IoT information in the subnet increased, the proposed technique of grouping and managing similar information to IoT information hierarchically obtained an average 12.9% lower overhead result. This result is due to improved accessibility of IoT information with probability-linked values and probabilistic weighting factors through the blockchain. Furthermore, the AIoT edge computing was hierarchically grouped into *n* subnets to classify IoT information so that no further operation could proceed.

#### 4.3.5. Evaluating IoT Connectivity to Validate IoT Integrity Based on Subnet Number

Table 7 shows connectivity for IoT devices deployed in the AIoT edge computing according to the number of subnet. In Table 7, the proposed technique requires linking probability values between IoT devices to minimize information errors in IoT devices. As a result of the performance evaluation, we found that the connectivity decreases by an average of 7.8% as the number of splits of the subnet increases by linking IoT devices together. These results are due to maintaining inter-IoT device linkage probability values to minimize information errors between IoT devices.

## 5. Conclusions

As IoT technology has recently been used in various fields, it has brought about many social changes and developments through IoT technology in cloud environments. In particular, as the cloud environment changes rapidly, servers that manage the cloud are also distributed and operated. However, safety issues are required for IoT devices used in cloud environments as many types of information are sent and received depending on cloud services. In this paper, a blockchain-based IoT big data integrity verification technique optimized for an AIoT edge computing environment was proposed. The proposed technique features information and signature keys of heterogeneous IoT devices contained in grouped subnets in the AIoT edge computing environment, creating multiple hash chains based on the blockchain to minimize IoT information loss. Servers in the subnet group guarantee the reliability of IoT integrity verification by connecting IoT information with hash values of each block to verify the blocks and deciding whether to add block information connected to the blockchain. As a result of performance evaluation, the proposed technique achieved an average of 13.7% improvement in IoT integrity verification time according to IoT blockchain generation probability value. The proposed technique resulted in up to 15.1% more efficient subnet gateway servers than when IoT information is linked to a blockchain depending on the size of the subnet. Furthermore, as the block size of IoT information increased based on the blockchain, the proposed technique resulted in an average 7.9% lower integrity verification delay. Finally, the proposed technique had an average 12.9% lower overhead of grouping and managing similar IoT information hierarchically as the number of IoT information in the subnet increased. Based on the results of this study, future research is planned on problems and supplementation by comparing and evaluating the integrity verification techniques of IoT information according to the types of AIoT edge computing services.

## Figures and Tables

**Table 1:** Classification of AIoT Implementation Methods.

Classification		Explanation	Example
Cloud intelligence utilize	Intelligent Cloud Platform Utilization	- Utilization of cognitive services such as vision, language, and machine learning services provided by cloud platforms of global IT companies such as Google, Amazon, IBM, MS, etc.	- Amazon Alexa - Self-driving cars up - Robot Pepper
	Intelligent IoT Services Cloud Platform Utilization	- Provide intelligent IoT services by adding recognition and analysis capabilities to the service cloud platform built by hardware object manufacturers to provide applied services	- Artificial Intelligence Home Appliances (LG, Samsung) - Interactive Secretary (Bixby, Siri, etc.)
Intelligence of things	Intelligent Engine Mounting Things on board	- Intelligent engine based on learning algorithms (machine learning, deep learning, etc.) is equipped with its own cognitive and thinking capabilities	- Nest Thermostat - MIT Boxster Robot
	Intelligent Object Platform and Cognitive Tools Utilization	- Utilization of object platforms to be mounted on objects requiring specialized intelligence, such as data analysis and self-driving cars	- IBM Quark - Qualcomm Drive - Data Platform

**Table 2:** Environment setup.

Parameter	Value
The number of server	1
The number of AIoT	20
The number of IoT	300
The transmit/receive power of the $IoT_{U}$	0.15 W/0.1 W
The network coverage radius	500 m
The static circuit power $P_{C}$	0.03W
The path loss exponent ε	3
The subnet tree depth	5
The available bandwidth for $\beta_{S}$/$\beta_{IoT}$	10 MHz/5 MHz
The power of noise $\pi^{2}$	−174 dBm/Hz
Subnet storage capacity	1 TB
Input data size $D_{m,\;n}$	3 kbits/s
Delay threshold $\tau_{m,\;n}$	10 s
Link capacity $L_{m,n}$	10 Gbps
Poisson lambda	80%
Data generation span	10 min
Max access count	100
The unit price of energy $\varphi_{e}$	0.15 Token/J

**Table 3:** IoT integrity verification time by blockchain generation probability value. Units: ms.

Value	Z. Yang et al.	C. Esposito et al.	D. He et al.	Proposed Scheme
1	17.525	15.572	14.505	12.942
2	16.805	16.141	14.779	13.153
3	18.167	15.723	14.807	12.769
4	17.428	16.534	15.376	13.195
5	16.943	15.756	14.374	12.935
6	18.732	16.142	15.625	13.548
7	18.285	16.655	15.386	12.474
8	17.229	15.481	14.508	12.921
9	18.295	16.038	15.478	13.295

**Table 4:** The Efficiency of IoT information processing in subnet gateway servers. Units: %.

Value	Z. Yang et al.	C. Esposito et al.	D. He et al.	Proposed Scheme
1	58.32	62.39	68.58	73.25
2	67.59	73.01	77.19	82.07
5	72.48	77.36	81.27	86.49
10	75.06	79.18	83.92	87.43
15	77.36	81.65	84.72	88.25
20	79.15	83.09	85.14	89.08

**Table 5:** Delay time for verification of the integrity of blockchain information. Units: ms.

Value	Z. Yang et al.	C. Esposito et al.	D. He et al.	Proposed Scheme
1	43.907	37.102	28.762	22.337
2	46.275	40.538	30.189	23.874
5	50.649	45.647	37.546	28.478
10	54.735	50.493	39.098	35.744
15	62.404	53.285	44.277	38.285
20	66.581	55.188	47.645	42.968

**Table 6:** Overhead of IoT integrity validation by number of subnet gateway servers. Units: %.

Value	Z. Yang et al.	C. Esposito et al.	D. He et al.	Proposed Scheme
1	9.581	7.889	5.658	4.478
2	12.474	9.084	7.379	5.387
5	13.189	11.745	8.391	6.278
10	14.453	13.676	11.254	9.668
15	16.876	15.285	13.387	10.493
20	18.297	16.719	15.297	12.341

**Table 7:** Evaluating IoT connectivity to validate IoT integrity based on subnet number. Units: %.

Subnet Number	IoT Data Connectivity			
	FP	RP	FRP	Proposed Scheme
2	81.636	85.195	89.949	92.321
4	73.693	80.699	84.686	88.789
6	65.452	71.593	75.768	80.476
8	58.345	63.874	70.837	73.468
10	49.067	54.938	56.105	61.302
12	41.524	47.834	51.852	59.407

FP: Full pairwise. RP: Random Pairwise. FRP: Full and Random Pairwise.

## Data Availability

Publicly available datasets were analyzed in this study. The data presented in this study are available on request from the corresponding author.

## References

[1] Zhao Z., Barijough K.M., Gerstlauer Z. (2018). DeepThings: Distributed Adaptive Deep Learning Inference on Resource-Constrained IoT Edge Clusters. IEEE Trans. Comput. Aided Des. Integr. Cricuits Syst..

[2] Chen Y., He J., Zhang X., Hao C., Chen D. Cloud-DNN: An Open Framework for Mapping DNN Models to Cloud FPGAs. Proceedings of the International Symposium on Field-Programmable Gate Arrays(FPGA).

[3] Ouaddah A., Elkalam A.A., Ouahman A.A. (2017). Towards a Novel Privacy-Preserving Access Control Model Based on Blockchain Technology in IoT. Europe and MENA Cooperation Advances in Information and Communication Technologies.

[4] Xu L.D., He W., Li S. (2014). Internet of Things in industries: A survey. IEEE Trans. Ind. Inform..

[5] Miller D. (2018). Blockchain and the Internet of Things in the industrial sector. IT Prof..

[6] Li Z., Wang D. (2019). Achieving One-Round Password-based Authenticated Key Exchange over Lattices. IEEE Trans. Serv. Comput..

[7] Xu H., Yu W., Liu X., Griffith D., Golmie N. On Data Integrity Attacks against Industrial Internet of Things. Proceedings of the 2020 IEEE Intl Conf on Dependable, Autonomic and Secure Computing, Intl Conf on Pervasive Intelligence and Computing, Intl Conf on Cloud and Big Data Computing, Intl Conf on Cyber Science and Technology Congress (DASC/PiCom/CBDCom/CyberSciTech).

[8] Xiong Z., Cai Z., Takabi D., Li W. (2021). Privacy Threat and Defense for Federated Learning with Non-i.i.d. Data in AIoT. IEEE Trans. Ind. Inform..

[9] Yu F.R., Liu J.M., He Y., Si P.B., Zhang Y.H. (2018). Virtualization for distributed ledger technology (VDLT). IEEE Access.

[10] Eyal I., Gencer A.E., Sirer E.G., Renesse R.V. Bitcoin-NG: A scalable blockchain protocol. Proceedings of the 13th USENIX Symposium Network System.

[11] Aitzhan N.Z., Svetinovic D. (2018). Security and privacy in decentralized energy trading through multi-signatures, blockchain and anonymous messaging streamsm. IEEE Trans. Dependable Secur. Comput..

[12] Rafael P., Elaine S. FruitChains: A Fair Blockchain. Proceedings of the ACM Symposium on Principles of Distributed Computing.

[13] Xu X., Weber I., Staples M., Zhu L., Bosch J., Bass L., Pautasso C., Rimba A. A Taxonomy of Blockchain-based Systems for Architecture Design. Proceedings of the IEEE International Conference on Software Architecture (ICSA17).

[14] Khaqqi K.N., Sikorski J.J., Hadinoto K., Kraft M. (2018). Incorporating seller/buyer reputation-based system in blockchain-enabled emission trading application. Appl. Energy.

[15] Yang Z., Zheng K., Yang K., Leung V.C. A blockchain-based reputation system for data credibility assessment in vehicular networks. Proceedings of the 2017 IEEE 28th PIMRC Symposium.

[16] Schaub A., Bazin R., Hasan O., Brunie L. A trustless privacy preserving reputation system. Proceedings of the IFIP International Information Security and Privacy Conference.

[17] Moinet A., Darties B., Baril J.-L. (2017). Blockchain based trust & authentication for decentralized sensor networks. arXiv.

[18] Karati A., Islam S.H., Biswas G.P., Bhuiyan M.Z.A., Vijayakumar P., Karuppiah M. (2018). Provably secure identity-based signcryption scheme for crowdsourced industrial Internet of things environments. IEEE Internet Things J..

[19] Esposito C., Castiglione A., Palmieri F., Santis A.D. (2018). Integrity for an event notification within the industrial Internet of things by using group signatures. IEEE Trans. Ind. Inform..

[20] He D., Kumar N., Choo K.R., Wu W. (2017). Efficient hierarchical identity-based signature with batch verification for automatic dependent surveillance-broadcast system. IEEE Trans. Inf. Forensics Secur..

[21] Bouachir Q., Aloquaily M., Tesng L., Boukerche A. (2020). Blockchain and Fog Computing for Cyberphysical Systems: The Case of Smart Industry. Computer.

[22] Dong G., Wang X. A Secure IoT Data Integrity Auditing Scheme Based on Consortium Blockchain. In Proceedings of the 2020 5th IEEE International Conference on Big Data Analytics(ICBDA).

[23] Urien P. Proving IoT Devices Firmware Integrity with Bijective MAC Time Stamped. Proceedings of the 2020 IEEE 6th World Forum on Internet of Things(WF-IoT).

[24] Chen Y.J., Wang L.C., Wang S. (2020). Stochastic Blockchain for IoT Data Integrity. IEEE Trans. Netw. Sci. Eng..

[25] Cherupally S.R., Boga S., Podili P., Kataoka K. Lightweight and Scalable DAG based distributed ledger for verifying IoT data integrity. Proceedings of the 2021 International Conference on Information Networking (ICOIN).

[26] Kuo S.S., Su W.T. A Blockchain-Indexted Storage supporting Scalable Data Integrity in Supply Chain Traceability. Proceedings of the 2020 IEEE International Conference on Smart Internet of Things(SmartIoT).

[27] Morton A. (2009). Performance Metrics for All. IEEE Internet Comput..

